# Depression: an individual-level early warning indicator of virologic failure in HIV patients in South Africa

**DOI:** 10.5588/pha.24.0017

**Published:** 2024-06-01

**Authors:** J.A. Edwards, J. Brijkumar, M. Dudgeon, C. Robichaux, B. Johnson, L. Rautman, R.A. Powers, Y.V. Sun, S. Pillay, C. Ordonez, J. Castillo-Mancilla, F.C. Tanser, Z. Asghar, P. Mee, P. Moodley, H. Sunpath, D.R. Kuritzkes, V.C. Marconi, M-Y.S. Moosa

**Affiliations:** ^1^ Department of Biostatistics and Bioinformatics Rollins School of Public Health, Emory University, Atlanta, GA, USA;; ^2^ University of Lincoln School of Health and Science, Lincoln, UK;; ^3^ Nelson R. Mandela School of Medicine, University of KwaZulu-Natal, Durban, KwaZulu-Natal, South Africa;; ^4^ Department of Medicine, Emory University School of Medicine, Atlanta, GA,; ^5^ Department of Biomedical Informatics, Emory University School of Medicine, Atlanta, GA,; ^6^ Department of Biostatistics and Computation Biology, University of Rochester, Rochester, NY, USA;; ^7^ Infectious Disease Epidemiology, Bernhard-Nocht Institute for Tropical Medicine, Hamburg, Germany;; ^8^ Department of Global Health, Rollins School of Public Health, Emory University, Atlanta, GA,; ^9^ Department of Epidemiology, Rollins School of Public Health, Atlanta, GA, USA;; ^10^ Adrenergy Research Innovations, Durban, KwaZulu-Natal, South Africa;; ^11^ Division of Infectious Diseases, Department of Medicine, University of Colorado, Aurora, CO, USA;; ^12^ Centre for Epidemic Response and Innovation, School for Data Science and Computational Thinking, Stellenbosch University, Stellenbosch,; ^13^ Africa Health Research Institute, KwaZulu-Natal,; ^14^ Department of Virology, National Health Laboratory Service and School of Laboratory Medicine and Medical Sciences, University of KwaZulu-Natal Durban, South Africa;; ^15^ Division of Infectious Diseases, Brigham and Women's Hospital, Boston, MA,; ^16^ Department of Medicine, Harvard Medical School, Boston, MA, USA

**Keywords:** mental health, viral load, risk factors, treatment failure

## Abstract

**OBJECTIVE:**

To identify individual-level early warning indicators of virologic failure in HIV patients receiving antiretroviral therapy (ART) in South Africa.

**DESIGN:**

A matched case–control study of individuals with and without virologic failure (VF) (>5 months on ART and HIV-1 plasma viral load >1,000 copies/mL) was conducted between June 2014 and June 2018. Of the 1,000 participants enrolled in the parent cohort, 96 experienced VF, and 199 additional controls were identified from the parent cohort and matched 1:2 (some matched 1:3) for sex, age, ART duration, and site. Participants were interviewed while clinical, pharmacy refill, laboratory, and objective pharmacological data were obtained. Multivariate conditional logistic regression models were constructed using model selection to identify factors associated with VF. Significant determinants of VF were identified using an alpha level of 0.05.

**RESULTS:**

In a full conditional model, higher cumulative ART adherence, quantified using tenofovir-diphosphate concentrations in dried blood spots (OR 0.26) and medication possession ratio (OR 0.98) were protective against VF, whereas an increase in total depression score (OR 1.20) was predictive of VF.

**CONCLUSION:**

This analysis demonstrates the importance of depression as a key individual-level early warning indicator of VF. Efforts to address mental health concerns among patients with people living with HIV could improve virologic suppression.

## Abstract (fr)

**OBJECTIF:**

Identifier les indicateurs d'alerte précoce au niveau individuel de l'échec virologique chez les patients séropositifs recevant un traitement antirétroviral (TAR) en Afrique du Sud.

**MÉTHODE:**

Une étude cas-témoins appariée de personnes avec et sans échec virologique (FV, pour l’anglais « *virologic failure* ») (>5 mois sous ART et charge virale plasmatique du VIH-1 >1 000 copies/ml) a été menée entre juin 2014 et juin 2018. Sur les 1 000 participants inscrits dans la cohorte parente, 96 ont présenté une FV et 199 témoins supplémentaires ont été identifiés dans la cohorte parentale et appariés 1:2 (certains appariés 1:3) pour le sexe, l'âge, la durée du TAR et le site. Les participants ont été interrogés pendant que des données cliniques, de renouvellement de pharmacie, de laboratoire et pharmacologiques objectives ont été obtenues. Des modèles de régression logistique conditionnelle multivariée ont été construits à l'aide d'une sélection de modèles pour identifier les facteurs associés à la FV. Les déterminants significatifs de la FV ont été identifiés à l'aide d'un niveau alpha de 0,05.

**RÉSULTATS:**

Dans un modèle conditionnel complet, une observance cumulative plus élevée du TAR, quantifiée à l'aide des concentrations de ténofovir-diphosphate dans les gouttes de sang séché (OR 0,26) et du ratio de possession de médicaments (OR 0,98) protégeait contre la FV, tandis qu'une augmentation du score de dépression totale (OR 1,20) était prédictive de la FV.

**CONCLUSION:**

Cette analyse démontre l'importance de la dépression en tant qu'indicateur précoce clé au niveau individuel de la FV. Les efforts visant à résoudre les problèmes de santé mentale chez les personnes vivant avec le VIH pourraient améliorer la suppression virologique.

After more than four decades of the global HIV pandemic, 84.2 million people have been diagnosed with HIV infection, and over half of these individuals are alive because of life-saving antiretroviral therapy (ART). Quantitative plasma HIV-1 RNA viral load (VL) testing remains the gold standard for monitoring ART response in patients with HIV (PWH).^1^ Sustained VL suppression greatly reduces the risks of drug resistance, disease progression, and mortality, whereas virologic failure (VF) can compromise future treatment options and contribute to ongoing viral transmission. In South Africa, VF is defined as two consecutive VL measurements of >1,000 copies/mL, separated by two to three months of enhanced adherence counseling. The causal web surrounding VF is highly centered on adherence-dependent factors; however, recent research suggests that a more complex nexus of factors should be considered.^2–5^ Distal factors, such as income, community, and familial ties, also play an important role in complex interactions beyond just biology^2–5^. The causes of VF (adherence, absorption, and resistance) are ultimately influenced by complex medical, personal, and community interactions.^6,7^

The WHO recommends monitoring early warning indicators (EWI) for the emergence of drug resistance at the population level.^8^ EWI identifies which programs, districts, or regions are at risk of drug resistance due to suboptimal adherence, ART shortages, or clinic retention.^5,8^ Ideally, EWI could be developed at the individual level to identify VF even in the absence of VL data or genotype resistance testing.^9,10^

As the clinical care of PWH transitions into a highly manageable chronic disease demanding durable adherence and regular but infrequent monitoring, the development of instruments to risk stratify individuals at risk for ART failure and resistance has become urgent and critical. The primary aim of the KwaZulu-Natal (KZN) HIV AIDS Drug Resistance Surveillance Study (ADReSS) was to identify candidate individual-level factors that might serve as EWI of VF in South Africa.

## METHODS

### Clinical setting

The parent study for this sub-analysis, ADReSS, was conducted at two clinical sites, with methods and results described in detail elsewhere.^11,12^ Bethesda Hospital is a rural district hospital with an attached clinic and two satellite clinics located in the northeastern KZN province close to the borders of Mozambique and Eswatini. RK Khan Hospital is a large, peri-urban state-run regional hospital located 20 km south of Durban, KZN, South Africa. After receiving and signing informed consent, all recruited participants in these clinics underwent VL testing performed at 6 and 12 months after ART initiation, followed by annual testing thereafter for those individuals virologically suppressed as part of the standard of care. For participants with VF, follow-up VL testing was performed 2–3 months after enhanced adherence counseling to confirm successful re-suppression. If VF persisted, the regimen was changed to a second-line ART.

### Study design and participants

ADReSS was a nested, matched case–control study (1:2), with a small proportion of the subjects matched 1:3. Cases were identified from the prospective cohort if they experienced a single VL >1,000 copies/mL within 1–2 weeks of a visit to the clinic when blood was drawn (which corresponded to a pharmacy refill visit) by the study clinician. Controls had all VL <1,000 copies/mL and were matched by age group, sex, site, and ART duration.

Each site enrolled 500 ART-naïve patients into the parent study from June 2014 to June 2018 who were initiated on first-line treatment with efavirenz/tenofovir disoproxil fumarate/lamivudine, as previously described. Of these recruited participants, 96 experienced VF and were matched with 196 controls. The analytical data set included a matched case/control group of 294 participants. The CONSORT (Consolidated Standards of Reporting Trials) diagram can be found in (Figure).

**FIGURE.**
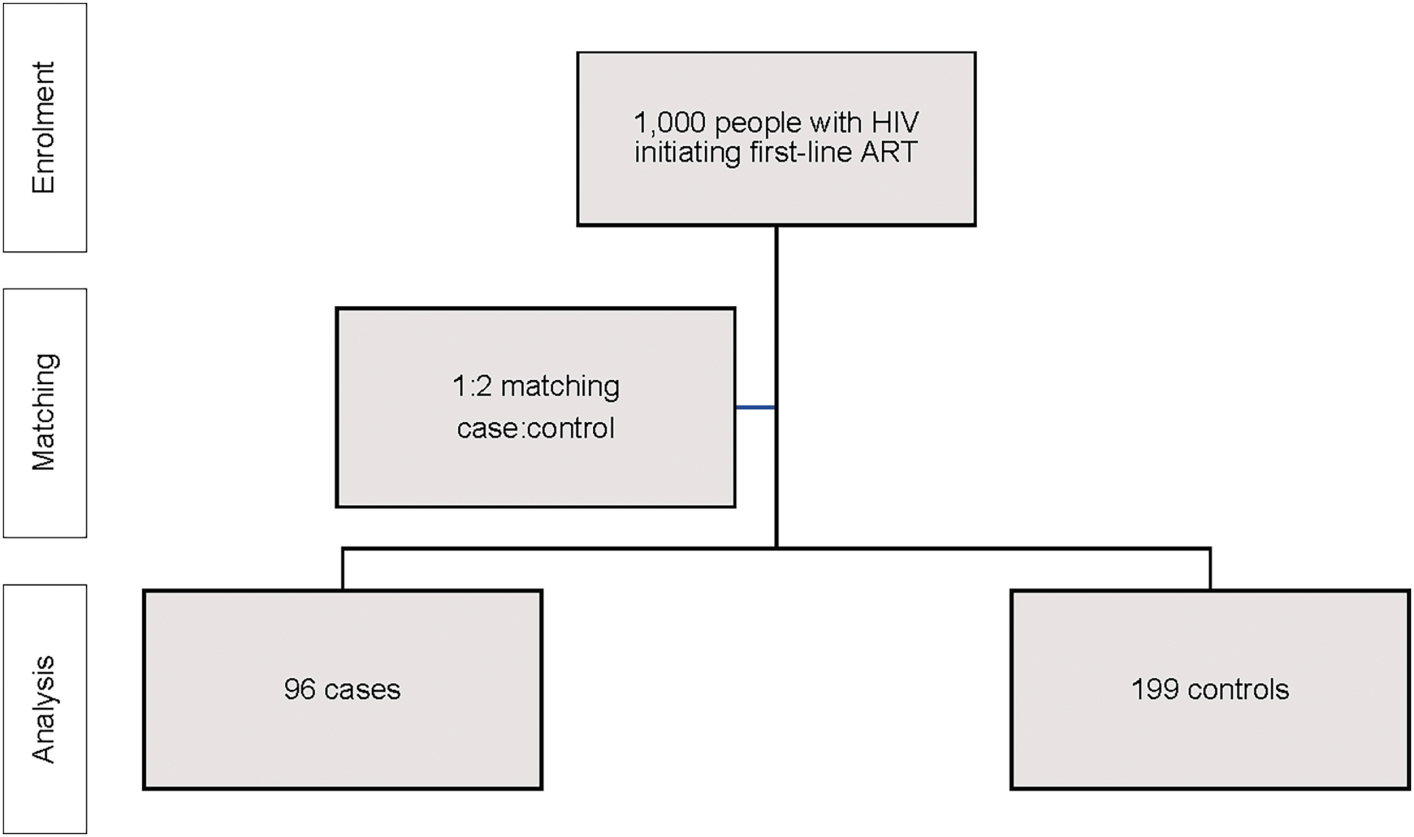
CONSORT (Consolidated Standards of Reporting Trials) diagram. ART = antiretroviral therapy.

### Data collection

Cases and controls underwent a follow-up interview in their preferred language with a trained research assistant/clinical study nurse who was blinded to the case/control status. This interview comprised a questionnaire, validated neurocognitive assessment, and the Kessler 10 (K-10) depression scale.^13^ The questionnaire contained demographic, socioeconomic (including a wealth index,^3,5^ employment, education and cohabitants), psychosocial (including substance abuse, food insecurity, traditional African medicine use, safe sex practices, faith, stigma and intimate partner violence), and clinic satisfaction indices. There were also specific questions about ART adherence and clinic attendance based on the modified AIDS Clinical Trials Group (ACTG) adherence questionnaire.^14^ Study physicians met each participant to review their medical history, administer the symptom screen, and obtain the Karnofsky score.^15^ Clinical information, pharmacy refill dates/quantities, and laboratory data were abstracted from medical records and entered a case report form. Further details of the collected data can be found in Table 1. In addition, dried blood spots were obtained to quantify tenofovir-diphosphate (TFV-DP) levels, a clinically relevant measure of cumulative ART adherence, as previously described.^16^

**TABLE 1.** Baseline characteristics.

	Case (*n* = 96)	Control (*n* = 199)	Overall (*n* = 295)
	*n* (%)	*n* (%)	*n* (%)
Tenofovir level			
Mean ± SD	5.66 ± 1.53	6.55 ± 0.642	6.26 ± 1.09
Median (min–max)	6.11 (2.53 to 8.09)	6.62 (2.53 to 8.65)	6.55 (2.53 to 8.65)
Missing	4 (4.2)	6 (3.0)	10 (3.4)
Total depression score			
Mean ± SD	14.6 ± 5.36	12.3 ± 4.40	13.0 ± 4.85
Median (min–max)	13.0 (10.0 to 37.0)	11.0 (10.0 to 47.0)	11.0 (10.0 to 47.0)
Missing	2 (2.1)	7 (3.5)	9 (3.1)
What languages do you understand?			
English	4 (4.2)	3 (1.5)	7 (2.4)
Multilingual/other	26 (27.1)	54 (27.1)	80 (27.1)
Zulu	11 (11.5)	17 (8.5)	28 (9.5)
Zulu, English	55 (57.3)	125 (62.8)	180 (61.0)
Number of biological children			
Mean ± SD	2.43 ± 2.06	2.04 ± 1.65	2.16 ± 1.80
Median (min–max)	2.00 (0 to 11.0)	2.00 (0 to 9.00)	2.00 (0 to 11.0)
Missing	2 (2.1)	4 (2.0)	6 (2.0)
Highest grade completed			
Mean ± SD	9.42 ± 3.49	10.1 ± 2.50	9.89 ± 2.87
Median (min–max)	11.0 (0 to 12.0)	11.0 (0 to 12.0)	11.0 (0 to 12.0)
Years beyond matriculation			
None	79 (82.3)	160 (80.4)	239 (81.0)
Some	17 (17.7)	39 (19.6)	56 (19.0)
Do you receive other forms of support?			
No	50 (52.1)	100 (50.3)	150 (50.8)
Yes	44 (45.8)	96 (48.2)	140 (47.5)
Missing	2 (2.1)	3 (1.5)	5 (1.7)
Medication adherence, %			
Mean ± SD	66.7 ± 39.3	88.6 ± 22.5	81.5 ± 30.7
Median (min–max)	84.0 (0 to 117)	96.0 (0 to 146)	95.0 (0 to 146)
Missing	1 (1.0)	0 (0)	1 (0.3%)
Wealth Index 1			
Mean ± SD	–0.223 ± 2.06	0.0868 ± 1.80	–0.0125 ± 1.89
Median (min–max)	0.226 (–5.92 to 3.62)	0.226 (–7.73 to 3.62)	0.226 (–7.73 to 3.62)
Missing	7 (7.3)	10 (5.0)	17 (5.8)
Wealth Index 2			
Mean ± SD	0.189 ± 1.92	–0.0780 ± 1.60	0.00751 ± 1.71
Median (min–max)	–0.654 (–1.14 to 7.23)	–0.755 (–1.14 to 6.44)	–0.755 (–1.14 to 7.23)
Missing	7 (7.3)	10 (5.0)	17 (5.8)
Clinic travel time, min			
<30	28 (29.2)	53 (26.6)	81 (27.5)
>30	66 (68.8)	142 (71.4)	208 (70.5)
Missing	2 (2.1)	4 (2.0)	6 (2.0)
Any food insecurity in the last 4 weeks			
Never	69 (71.9)	146 (73.4)	215 (72.9)
Some	20 (20.8)	44 (22.1)	64 (21.7)
Missing	7 (7.3)	9 (4.5)	16 (5.4)
Current living arrangement			
Not with family	56 (58.3)	116 (58.3)	172 (58.3)
Stay with family	38 (39.6)	78 (39.2)	116 (39.3)
Missing	2 (2.1)	5 (2.5)	7 (2.4)
Housing (house/other)			
House	74 (77.1)	156 (78.4)	230 (78.0)
Other	20 (20.8)	40 (20.1)	60 (20.3)
Missing	2 (2.1)	3 (1.5)	5 (1.7)
Fully employed			
Full employment	27 (28.1)	62 (31.2)	89 (30.2)
Less than full employment	67 (69.8)	134 (67.3)	201 (68.1)
Missing	2 (2.1)	3 (1.5)	5 (1.7)
Unemployed			
Not unemployed	55 (57.3)	137 (68.8)	192 (65.1)
Unemployed	41 (42.7)	62 (31.2)	103 (34.9)
Care duration			
Mean ± SD	26.2 ± 13.7	23.9 ± 12.2	24.7 ± 12.8

SD = standard deviation.

The study data were managed using REDCap (Vanderbilt University, Nashville, TN, USA) electronic data capture tools hosted at Emory University, Atlanta, GA, USA.^17,18^

### Statistical analysis

Data were analyzed using RStudio v.4.1717 (Posit PBC, Boston, MA, USA). The medication possession ratio (MPR) was calculated for all participants using previously reported methods.^5,19^ Stratified conditional logistic regression was used throughout the analysis, with matched sets defined using the five aforementioned matching variables and the primary outcome of VF defining the case-control status.^20^ Missingness was assessed, and variables with >15% missing values were excluded from the analysis. All variables were independently analyzed via stratified conditional logistic regression for their association with the primary outcome using univariate Wald tests. Although all variables were examined, only those meeting significance (*P* < 0.05) in univariate analyses and any variable considered epidemiologically relevant were analyzed further. Several multivariate stratified conditional logistic regression models were constructed using model selection to arrive at the final models. Model fit was assessed using the Akaike Information Criterion (AIC), and the results are shown in Table 2.

**TABLE 2.** Model results.

	Only baseline variables		Full mode		Full without tenofovir/medication adherence		Full without tenofovir	
Coefficient	Estimates (95% CI)	*P* value	Estimates (95% CI)	*P* value	Estimates (95% CI)	*P* value	Estimates (95% CI)	*P* value
Understand language (multilingual/other)	0.59 (0.10‒3.45)	0.555	0.29 (0.02–5.43)	0.406	0.55 (0.09–3.58)	0.533	0.71 (0.09–5.44)	0.745
Understand language (Zulu)	0.51 (0.07-3.89)	0.515	0.41 (0.02–8.54)	0.569	0.49 (0.06–4.13)	0.509	0.78 (0.07–8.44)	0.835
Understand language (Zulu/English)	0.48 (0.09–2.62)	0.393	0.14 (0.01–2.00)	0.146	0.37 (0.06–2.28)	0.285	0.45 (0.06–3.27)	0.433
Number of biological children	1.17 (0.97–1.42)	0.097	1.04 (0.73–1.47)	0.833	1.09 (0.89–1.33)	0.399	1.05 (0.84–1.32)	0.649
Highest grade completed	0.92 (0.80–1.07)	0.282	1.03 (0.84–1.27)	0.761	0.95 (0.82–1.10)	0.500	0.96 (0.81–1.14)	0.673
Years beyond matriculation	0.73 (0.32–1.65)	0.444	0.38 (0.10–1.49)	0.166	0.56 (0.23–1.37)	0.206	0.65 (0.24–1.76)	0.394
Received other support	0.60 (0.29–1.26)	0.179	0.71 (0.22–2.30)	0.564	0.66 (0.30–1.44)	0.295	0.74 (0.30–1.84)	0.516
Wealth Index 1	0.92 (0.74–1.15)	0.485	0.98 (0.73–1.32)	0.887	0.93 (0.74–1.17)	0.557	0.96 (0.76–1.23)	0.774
Wealth Index 2	1.10 (0.91–1.32)	0.318	1.19 (0.91–1.55)	0.194	1.13 (0.92–1.39)	0.237	1.16 (0.93–1.46)	0.195
Clinic >30 min from home	0.75 (0.37–1.50)	0.416	0.69 (0.23–2.08)	0.504	0.72 (0.33–1.58)	0.416	0.67 (0.28–1.61)	0.367
Food insecurity (some)	0.67 (0.26–1.72)	0.400	0.77 (0.20–2.89)	0.695	0.58 (0.22–1.54)	0.272	0.75 (0.24–2.34)	0.619
Lives with family	1.21 (0.61–2.40)	0.595	0.92 (0.32–2.60)	0.871	1.25 (0.58–2.68)	0.568	1.37 (0.60–3.12)	0.454
Lives in a house	0.77 (0.32–1.87)	0.564	0.45 (0.13–1.60)	0.219	0.53 (0.21–1.39)	0.199	0.56 (0.20–1.53)	0.258
Full employment (less than full employment)	1.03 (0.45–2.37)	0.946	0.93 (0.28–3.07)	0.910	1.06 (0.43–2.58)	0.900	1.09 (0.43–2.76)	0.861
Unemployed	2.09 (0.92–4.76)	0.080	1.37 (0.39–4.86)	0.622	1.43 (0.60–3.42)	0.420	1.08 (0.40–2.94)	0.878
Tenofovir level (log)			0.26 (0.12–0.56)	0.001				
Depression Score (total)			1.20 (1.07–1.34)	0.002	1.17 (1.07–1.28)	<0.001*	1.18 (1.07–1.31)	0.001*
Medication adherence, %			0.98 (0.96–1.00)	0.025*			0.97 (0.96–0.99)	<0.001*
Observations	267		253		264		263	
*R*^2^ Nagelkerke	0.123		0.562		0.233		0.379	
AIC	197.357		122.623		181.159		159.017	

*Statistically significant.

CI = confidence interval; AIC = Akaike Information Criterion.

Model 1 attempted to identify the factors underlying socioeconomic variables that were present at enrollment and unaffected by study participation (underlying factors). Model 2 included all statistically significant and epidemiologically relevant variables. To investigate whether adherence factors had an outsized effect on model performance, Models 3 and 4 were created; they were similar to Model 2 but without the presence of adherence factors.

### Ethics

The ADReSS study was approved by the Biomedical Research Ethics Committee at the University of KwaZulu-Natal, Durban, KwaZulu-Natal, South Africa, and by the Institutional Review Boards at Emory University, Atlanta, GA, USA; and Mass General Brigham in Boston, MA, USA.

## RESULTS

### Cohort description

The significant demographic and epidemiological characteristics of the study population are presented in Table 1. Overall, the mean age of the study population at the beginning of treatment was 32 years, 59% were women, and 96% identified as Black. Among all participants, 9.5% reported only understanding Zulu, while 61% reported understanding both Zulu and English. Economically, 68% considered themselves less than fully employed, 35% were unemployed, and 48% reported receiving other forms of support. Participants had a mean of 9.9 years of education and a median of two children living with them. Seventy percent of participants reported living more than 30 min away from the clinic, and 22% had at least some food insecurity in the 4 weeks before the study visit. More than 39% of the respondents were living with their family at the time of the survey, and 78% were living in a house. MPR averaged 82%, and TFV-DP concentrations in dried blood spot punch log values averaged 6.26 fmol/punch. The median total K-10 depression score was 11, and the mean duration of treatment was 25 months (standard deviation [SD] 12.8).

### Baseline risk factors (Model 1)

Due to the matching algorithm, age, sex, and duration of treatment were removed from the model. None of the variables analyzed were identified as risk factors for VF across all models (Table 2). For the variables present only at enrollment, none remained significant predictors of VF after adjustment.

### Full conditional model (Model 2)

With all statistically significant and epidemiologically relevant remaining variables included in the multivariate model (Table 2), the TFV-DP concentrations in the DBS (OR 0.26) and MPR (OR 0.98) variables were associated with a lower risk of VF. An increase in the total K-10 depression score (OR 1.20) was associated with VF. This model had the highest predictive value.

#### Full model without TFV-DP concentrations in DBS or MPR (Model 3)

Removing both TFV-DP in DBS and MPR (both measures of ART adherence) did not add any significant risk factors for VF, and an increase in the total depression score remained significant (OR 1.17).

#### Full model without TFV-DP concentration (Model 4)

TFV-DP in DBS was removed to determine whether this variable had an outsized influence on other variables (Table 2). After removal, we found no new significant predictors of VF, and an increase in the total K-10 depression score (OR 1.18) and MPR (OR 0.97) remained significant.

### Sensitivity and subgroup analysis

To determine the effectiveness of the matching algorithm, the variables used for matching (age, gender, and duration of ART) were stratified and added into the model with the matched variables removed one by one (Table 2). No new variables were significant in any of the four iterations. Within-group variation was also conducted on these four variables as well (Table 2). This procedure identified some differences among those above and below the median age, but none between sexes. ART duration and race/ethnicity analyses could not form complete models20.

## DISCUSSION

In this study, VF was most consistently associated with drug concentrations (TFV-DP) and the K-10 depression score, with some models also showing an association with medication pharmacy refill adherence (MPR).

Overall, despite an extensive survey, few risk factors were significantly associated with VF. This is distinct from other similar studies, both in sub-Saharan Africa and globally, which have found associations with factors such as marital status, occupation, and other economic and social factors considered in this study.

The association of TFV-DP concentrations and pharmacy refill adherence as protective against VF is reassuring as these factors are closest to the outcome. These findings are also consistent with those of prior studies on VF and biological models of proximate factors.^16,21^ Pharmacy refill adherence is likely to mediate serum levels of ART, whereas TFV-DP concentrations represent a direct point measure of ART serum levels.

PWH are more likely to develop depression, and depression is found in 20-32% of individuals with HIV.^22,23^ Although the association of depression score with VF has been demonstrated in some other studies,^5,24^ few studies have explored this association in depth. This association may be mediated by adherence or may be present even after controlling for medication adherence.^25,26^ This suggests that mental health conditions such as depression may mediate VF through both adherence-dependent and -independent mechanisms.

ADReSS was undertaken to extend the findings of the 2013 RFVF study, which investigated individual-level factors associated with VF for PWH receiving care in an urban KZN clinic.^5^ In that study, many variables, including male gender, not having an active religious faith, unprotected sex practices, having a family member with HIV infection, and not being pleased with the clinic experience, were associated with VF in- dependent of adherence measures. In contrast, in this study, only depression was a risk factor for VF. This suggests that depression, a risk factor for VF in both studies, may be a robust EWI and identify patients in need of psychological evaluation and treatment even in diverse settings.

The consistent association of elevated depression scores with VF across multiple models in this study and between studies suggests that, regardless of the proximal mechanisms involved, mental health conditions provide both an important aspect for consideration in the holistic management of PWH and a plausible individual-level EWI for VF. Consistent depression score monitoring should be considered as a clinical trigger for VL testing in populations of PWH whose VLs are less frequently monitored, or for earlier or more frequent VL monitoring. Another potential clinical intervention would be to prioritize mental health conditions (including efavirenz-induced) and the treatment of depression among PWH on ART, on par with other adherence measures. Although the K-10 and other depression scales are not diagnostic of Major Depressive Disorder, a positive screening test should prompt clinicians to refer patients for further evaluation and management.

## CONCLUSION

The complex causal web linking depression and VF suggested by this analysis argues for the inclusion of mental health for treating HIV. Rather than characterizing mental health as outside the purview of HIV care or as inappropriate diversions of funding better used for direct HIV care, such as ART or VL testing, this analysis suggests that goals such as virologic suppression and decreased AIDS mortality may in fact be stymied by not addressing factors such as depression. This may be particularly true in the South African context, which faces a high population prevalence of HIV and ongoing challenges in reaching UNAIDS targets, specifically 95% viral suppression in PWH on ART.^27,28^ The recently released “Reimagining PEPFAR Strategic Direction” document focuses on integrating HIV and non-communicable diseases care for PWH, with specific mention of filling gaps in service delivery, including person-centered care for mental health conditions.^29^

This study assessed several possible risk factors for VF, allowing for comprehensive modeling. Moreover, the collection of TFV-DP concentrations allows for direct measurement and modeling of a clear proximate cause of VF. However, this study relied on survey data collected from participants, and these data may have been affected by recall bias.

Despite variable matching, comprehensive data collection on potential risk factors, and robust multivariate modeling, only medication adherence and depression emerged as consistent, statistically significant predictors of VL. This replicates findings from a previous study focusing on an urban clinic in South Africa.^5^ These findings strengthen the assertion that two straightforward tools—the MPR and depression scale – can be used as individual-level EWI for VF. Moreover, both medication adherence and depression are amenable to intervention. Depression, in particular, is a key undertreated disease with significant morbidity, and its association with VF only adds to the need for its early detection and treatment.
